# Supplementary material for: Inorganic nanosheets facilitate humoral immunity against medical implant infections by modulating immune co-stimulatory pathways
Source: Nat Commun. 2022 Aug 18;13:4866. doi: 10.1038/s41467-022-32405-x (PMC9388665; doi:10.1038/s41467-022-32405-x)
Supplement: Supplementary file 1 — Supplementary Information [file 41467_2022_32405_MOESM1_ESM.pdf]

Supplementary Information

Inorganic nanosheets facilitate humoral immunity against medical implant infections by modulating immune co-stimulatory pathways

Chuang Yang†*, Yao Luo*†*, Hao Shen*†*, Min Ge, Jin Tang, Qiaojie Wang, Han Lin***, Jianlin Shi***, and Xianlong Zhang**

†Chuang Yang, Yao Luo and Hao Shen contributed equally to this manuscript.

Supplementary Figures

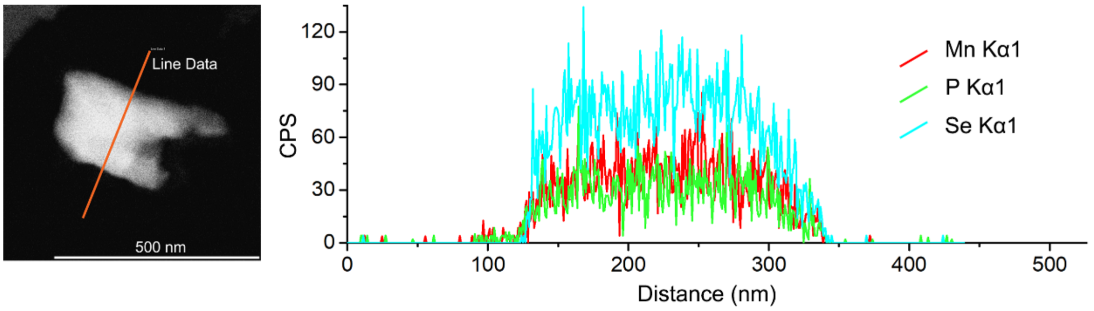

Supplementary Figure S1. Element-linear scanning of $MnPSe_3$ nanosheet.

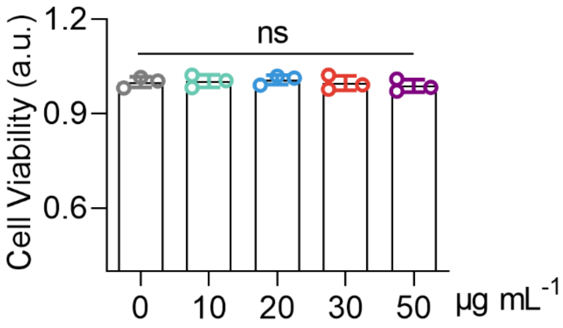

Supplementary Figure S2. Relative viabilities of MC3T3-E1 cells after being incubated with $MnPSe_3$-PVP at varied concentrations. Data are mean ± s.d. (n=3 per group) and n represents biologically independent experiments. One-way analysis of variance (ANOVA) with Tukey's post hoc test was used for multiple comparisons. ns, not significant. Source data are provided as a Source Data file.

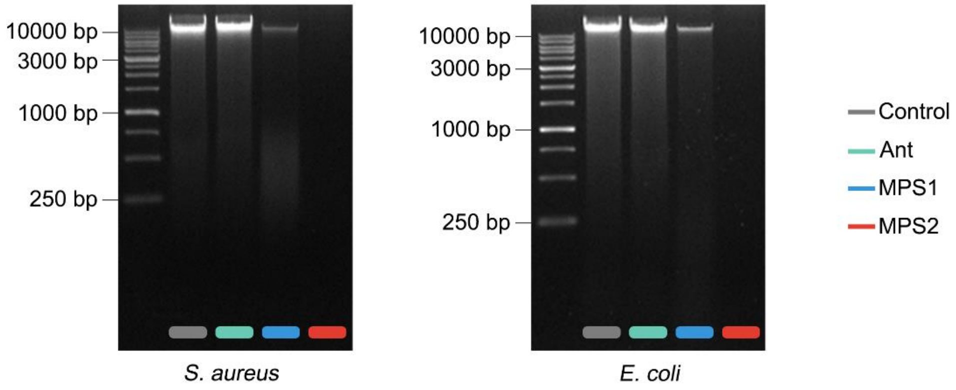

Supplementary Figure S3. Genomic DNA degradations of *S. aureus* and *E. coli* by different treatments. The experiments were repeated independently three times with similar results. Ant, abbreviation for antibiotics.

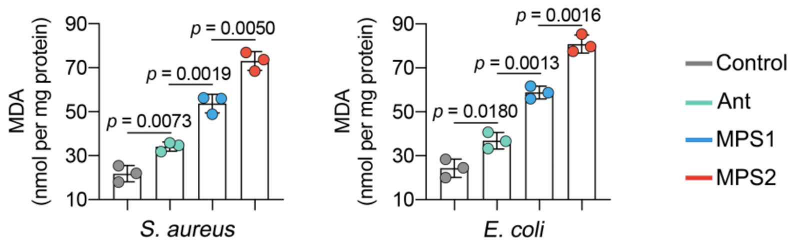

Supplementary Figure S4. Lipid peroxidations of *S. aureus* and *E. coli* with various treatments. Data are mean ± s.d. (n=3 per group) and n represents biologically independent experiments. Two-tailed, unpaired t-test, exact p-values. Ant, abbreviation for antibiotics. Source data are provided as a Source Data file.

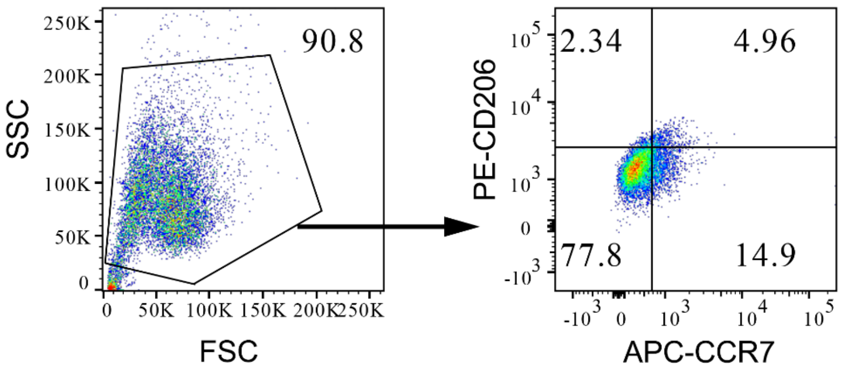

Supplementary Figure S5. Representative gating strategy of macrophages in vitro.

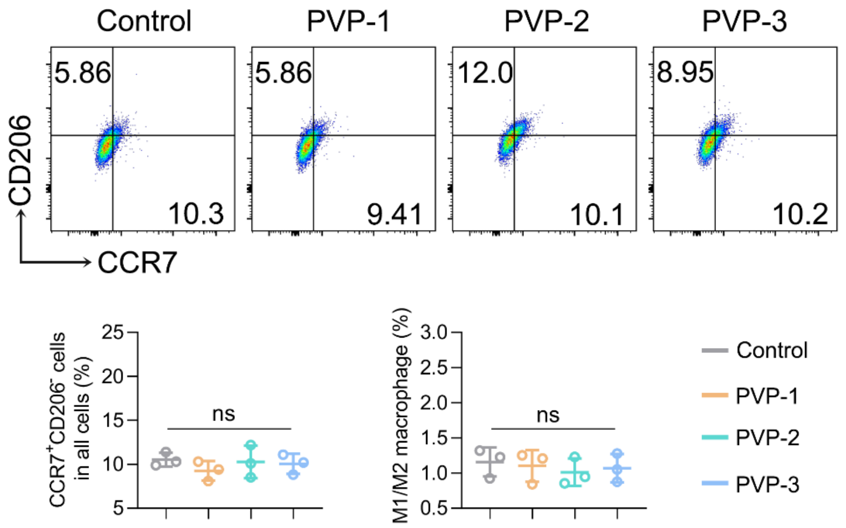

Supplementary Figure S6. Negative immune responses of macrophages by PVPs in vitro. Representative flow cytometry plots and quantification analysis showing the phenotype of macrophages (CCR7, M1 phenotype marker; CD206, M2 phenotype marker) after co-culture with different concentrations of PVPs (PVP-1, 10 μg mL^{-1}; PVP-2, 50 μg mL^{-1}; PVP-3, 100 μg mL^{-1}). Data are mean ± s.d. (n=3 per group) and n represents biologically independent experiments. One-way ANOVA with Tukey's post hoc test was used for multiple comparisons. ns, not significant. Source data are provided as a Source Data file.

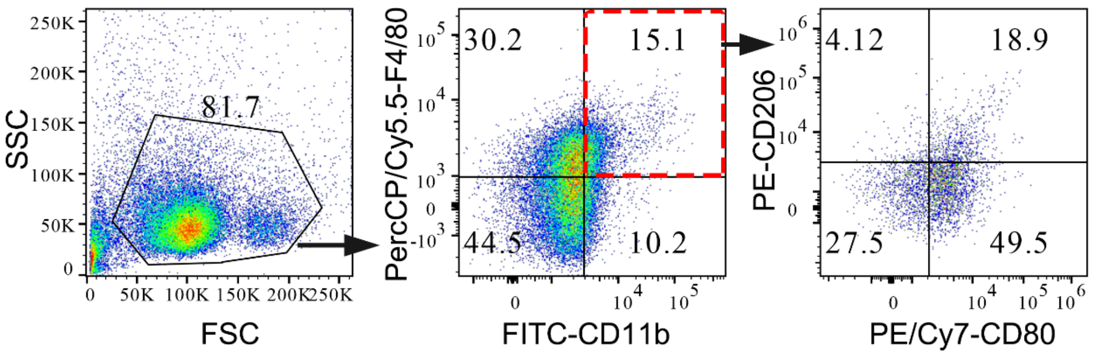

Supplementary Figure S7. Representative gating strategy of macrophages in IDLN of the mice "in situ" implant infection model.

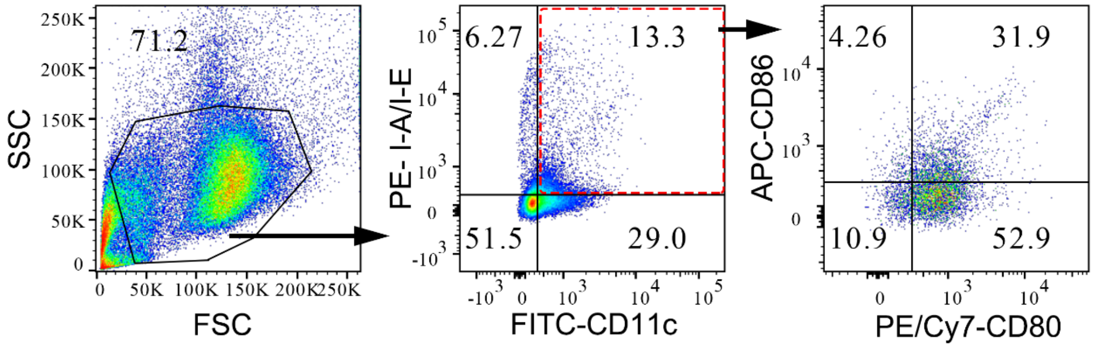

Supplementary Figure S8. Representative gating strategy of dendritic cells in IDLN of the mice "in situ" implant infection model.

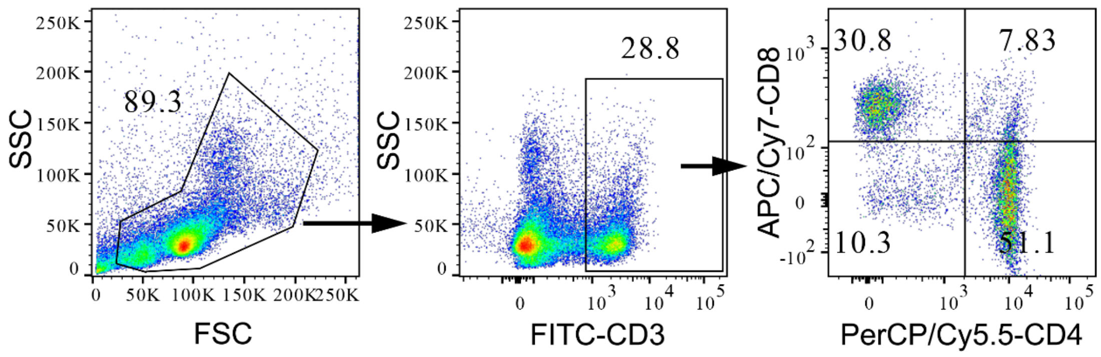

Supplementary Figure S9. Representative gating strategy of CD4$^+$ and CD8$^+$ T cells in IDLN of the mice "in situ" implant infection model.

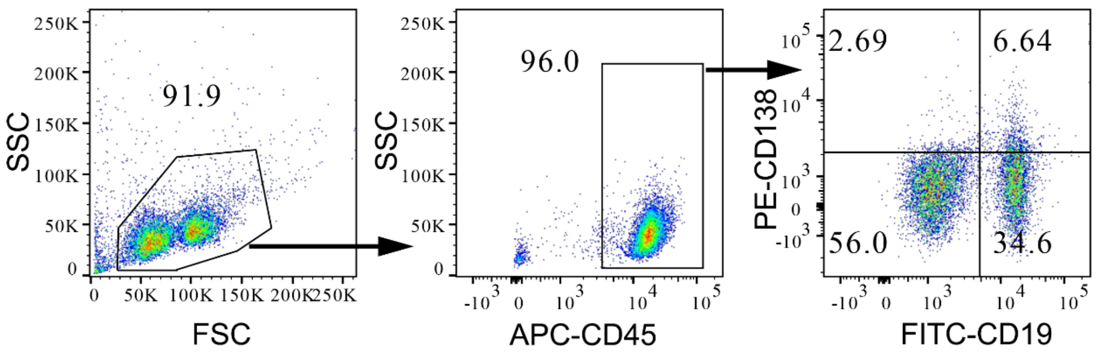

Supplementary Figure S10. Representative gating strategy of plasma cells and plasmablasts in IDLN of the mice "in situ" implant infection model.

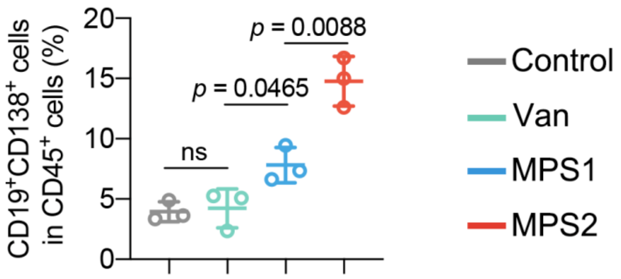

Supplementary Figure S11. Quantitative analysis of plasmablasts in IDLN of the mice "in situ" implant infection model. Data are mean ± s.d. (n=3 per group) and n represents biologically independent experiments. Two-tailed, unpaired t-test, exact p-values. ns, not significant. Van, abbreviation for vancomycin. Source data are provided as a Source Data file.

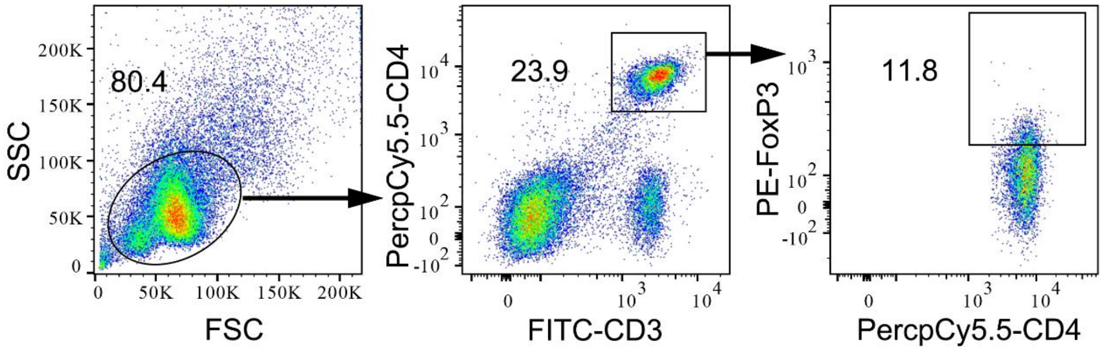

Supplementary Figure S12. Representative gating strategy of T_{reg}s in IDLN of the mice "in situ" implant infection model.

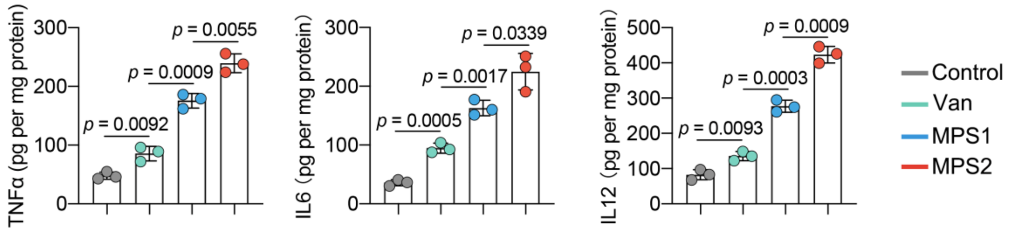

Supplementary Figure S13. Cytokine levels in infected tissues of the mice "in situ" implant infection model. Data are mean ± s.d. (*n*=3 per group) and *n* represents biologically independent experiments. Two-tailed, unpaired t-test, exact *p*-values. Van, abbreviation for vancomycin. Source data are provided as a Source Data file.

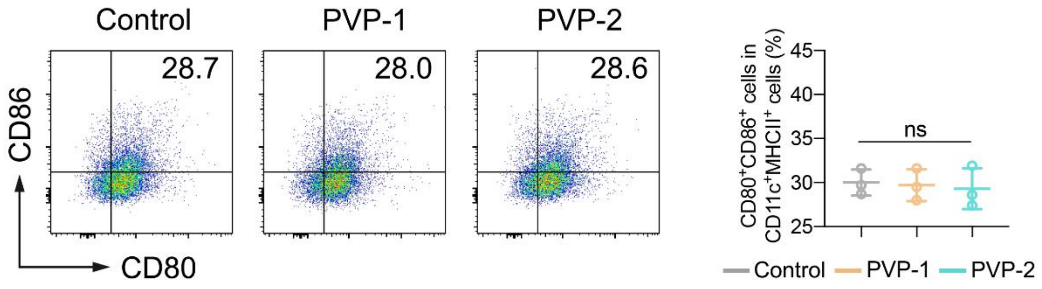

Supplementary Figure S14. Negligible influence of PVPs on dendritic cells (DCs) maturation in vivo. Flow cytometry analysis of mature DCs ($CD80^+CD86^+$ cells in $CD11c^+MHC\text{-}II^+$) in IDLN of the mice treated with different concentrations of PVPs (PVP-1, 10 mg kg^{-1}; PVP-2, 20 mg kg^{-1}) in the "in situ" implant infection model. Data are mean ± s.d. (*n*=3 per group) and *n* represents biologically independent experiments. One-way ANOVA with Tukey's post hoc test was used for multiple comparisons. ns, not significant. Source data are provided as a Source Data file.

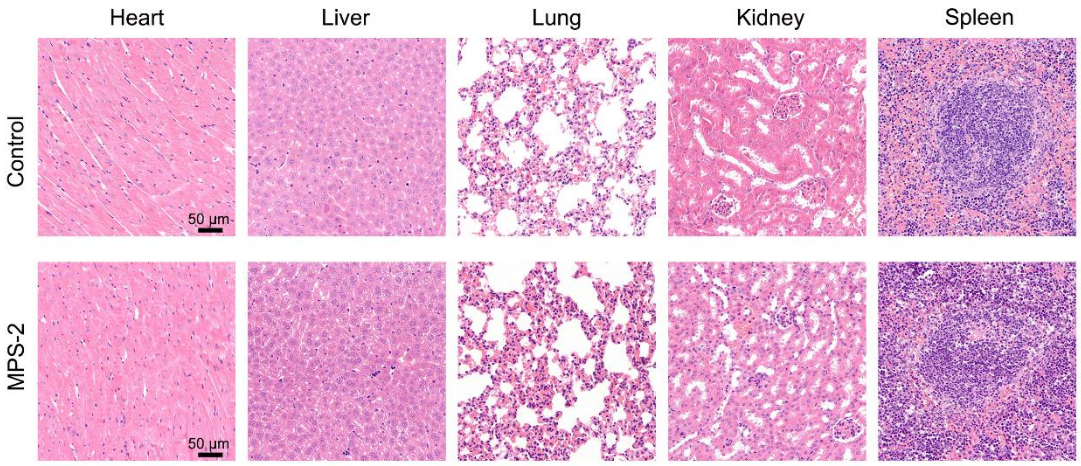

Supplementary Figure S15. Histological analysis of major organs after indicated treatments. Scale bar, 50 μm. Images are representative of three biologically independent mice.

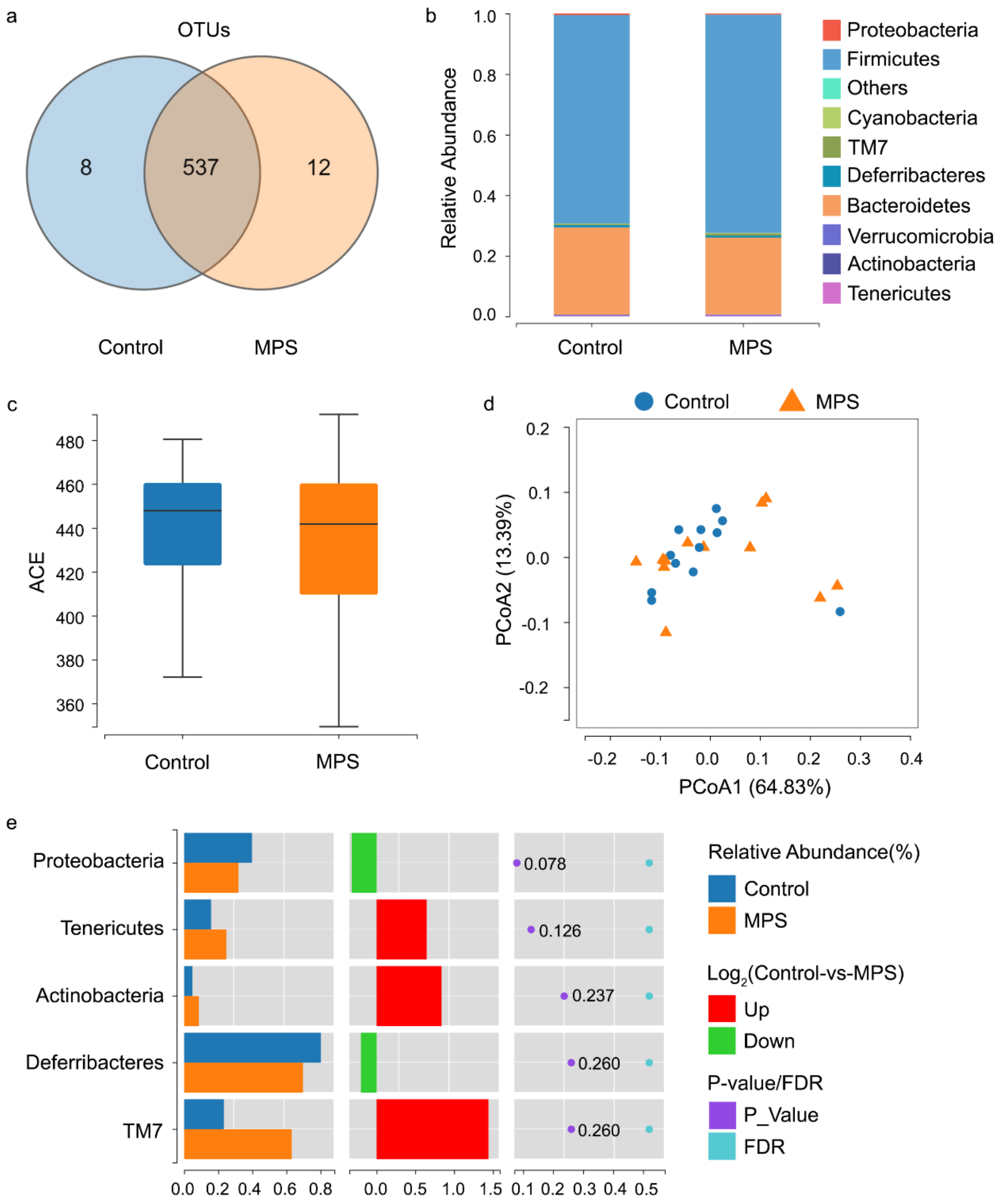

Supplementary Figure S16. 16S rRNA-targeted sequencing of gut microbiota of mice with "in situ" implant infection model after the MPS-PVP treatment. **a,** Venn diagram of detected bacterial OUTs (operational taxonomic units) in cecum contents of mice after treatment with PBS (Control) or MPS-PVP, respectively (12 mice per group). **b,** Taxonomic analysis at the phylum level of the gut microbiota from mice with different treatments. **c,** Alpha diversity was presented with ACE to compare the species richness of individual specimens. (*n*=12 biologically independent mice per

group. Box plot indicates median, 25th and 75th percentiles, and minima and maxima of the distribution.) **d,** Beta diversity was presented with principal coordinates analysis (PCoA) to reflect the species diversity in community composition and structure between different groups. **e,** Analysis of top 5 differentially abundant phylum in different groups. Two-sided, wilcox test, exact *p*-values. Benjamini-Hochberg (BH) test was used for multiple comparisons.

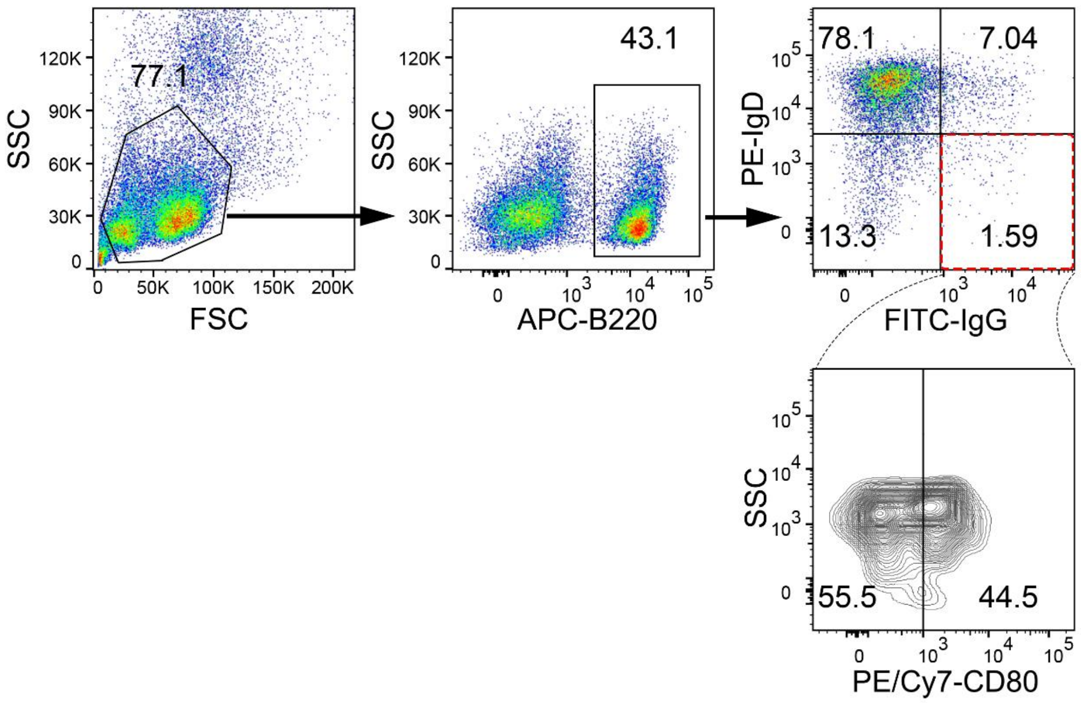

Supplementary Figure S17. Representative gating strategy of memory B cells and CD80$^+$ memory B cells in IDLN of the mice presurgical neoadjuvant immunotherapy model.

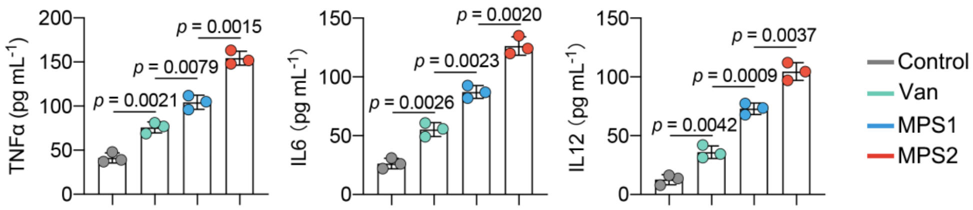

Supplementary Figure S18. Serum cytokine levels in the mice presurgical neoadjuvant immunotherapy model. Data are mean ± s.d. (n=3 per group) and n represents biologically independent experiments. Two-tailed, unpaired t-test, exact p-values. Van, abbreviation for vancomycin. Source data are provided as a Source Data file.

Supplementary Tables.

Supplementary Table 1. Antibody list for Fig.6.

**Color**	**Antibody**	**Clone**	**Company (Cat.#)**	**Dilution**
APC	CD45	30-F11	Biolegend (103111)	1:100
FITC	CD11b	M1/70	Biolegend (101205)	1:100
PerCP/Cy5.5	F4/80	BM8	Biolegend (123127)	1:100
PE/Cy7	CD80	16–10A1	Biolegend (104733)	1:100
PE	CD206	C068C2	Biolegend (141706)	1:100
FITC	CD11c	N418	Biolegend (117305)	1:100
PE	I-A/I-E	M5/114.15.2	Biolegend (107607)	1:100
APC	CD80	16–10A1	Biolegend (104713)	1:100
PE/Cy7	CD86	GL-1	Biolegend (105013)	1:100
FITC	CD3	17A2	Biolegend (100203)	1:100
APC/Cy7	CD8a	53–6.7	Biolegend (100714)	1:100
PerCP/Cy5.5	CD4	GK1.5	Biolegend (100433)	1:100
FITC	CD19	6D5	Biolegend (115505)	1:100
PE	CD138	281-2	Biolegend (142503)	1:100
PE	Foxp3	MF-14	Biolegend (126403)	1:100

Supplementary Table 2. Antibody list for Fig.7.

**Color**	**Antibody**	**Clone**	**Company (Cat.#)**	**Dilution**
APC	B220	RA3-6B2	Biolegend (103211)	1:100
PE	IgD	11-26c.2a	Biolegend (405705)	1:100
FITC	IgG	Poly4053	Biolegend (405305)	1:100
PE/Cy7	CD80	16–10A1	Biolegend (104733)	1:100
