# Supplementary material for: Inorganic nanosheets facilitate humoral immunity against medical implant infections by modulating immune co-stimulatory pathways
Source: Nat Commun. 2022 Aug 18;13:4866. doi: 10.1038/s41467-022-32405-x (PMC9388665; doi:10.1038/s41467-022-32405-x)
Supplement: Supplementary file 3 — Reporting Summary [file 41467_2022_32405_MOESM3_ESM.pdf]

nature portfolio

Corresponding author(s): Jianlin Shi

Last updated by author(s): Jul 15, 2022

Reporting Summary

Nature Portfolio wishes to improve the reproducibility of the work that we publish. This form provides structure for consistency and transparency in reporting. For further information on Nature Portfolio policies, see our Editorial Policies and the Editorial Policy Checklist.

Statistics

For all statistical analyses, confirm that the following items are present in the figure legend, table legend, main text, or Methods section.

n/a	Confirmed	
☐	☒	The exact sample size (*n*) for each experimental group/condition, given as a discrete number and unit of measurement
☐	☒	A statement on whether measurements were taken from distinct samples or whether the same sample was measured repeatedly
☐	☒	The statistical test(s) used AND whether they are one- or two-sided<br>*Only common tests should be described solely by name; describe more complex techniques in the Methods section.*
☒	☐	A description of all covariates tested
☐	☒	A description of any assumptions or corrections, such as tests of normality and adjustment for multiple comparisons
☐	☒	A full description of the statistical parameters including central tendency (e.g. means) or other basic estimates (e.g. regression coefficient) AND variation (e.g. standard deviation) or associated estimates of uncertainty (e.g. confidence intervals)
☐	☒	For null hypothesis testing, the test statistic (e.g. *F*, *t*, *r*) with confidence intervals, effect sizes, degrees of freedom and *P* value noted<br>*Give P values as exact values whenever suitable.*
☒	☐	For Bayesian analysis, information on the choice of priors and Markov chain Monte Carlo settings
☒	☐	For hierarchical and complex designs, identification of the appropriate level for tests and full reporting of outcomes
☒	☐	Estimates of effect sizes (e.g. Cohen's *d*, Pearson's *r*), indicating how they were calculated

Our web collection on statistics for biologists contains articles on many of the points above.

Software and code

Policy information about availability of computer code

Data collection	BD FACSDiva (1.00); Gatan DigitalMicrograph (3.7.4); Leica LAS X (3.5.5.19976). No custom code was used for data collection.
Data analysis	All statistical analyses were performed on Graphpad Prism 9 and Excel 2016. All the flow cytometry data were processed using FlowJo (v10; BD Biosciences, USA). 16S rRNA-targeted sequencing data are processed with RDP Classifier (v.2.2), Greengenes database (v201305), QIIME (v1.8.0) and MOTHUR (v1.31.2).

For manuscripts utilizing custom algorithms or software that are central to the research but not yet described in published literature, software must be made available to editors and reviewers. We strongly encourage code deposition in a community repository (e.g. GitHub). See the Nature Portfolio guidelines for submitting code & software for further information.

Data

Policy information about availability of data

All manuscripts must include a data availability statement. This statement should provide the following information, where applicable:

- Accession codes, unique identifiers, or web links for publicly available datasets
- A description of any restrictions on data availability
- For clinical datasets or third party data, please ensure that the statement adheres to our policy

The macrophage RNA-seq data have been deposited in NCBI Sequence Read Archive (SRA) database under the accession code PRJNA792491 (https://www.ncbi.nlm.nih.gov/sra/?term=PRJNA792491). The gut microbiota 16S rRNA-targeted sequencing data have been deposited in NCBI SRA database under the accession code PRJNA793390 (https://www.ncbi.nlm.nih.gov/sra/?term=PRJNA793390). The remaining data are available within the Article, Supplementary Information, or Source Data file. Source data are provided with this paper.

Field-specific reporting

Please select the one below that is the best fit for your research. If you are not sure, read the appropriate sections before making your selection.

☒ Life sciences ☐ Behavioural & social sciences ☐ Ecological, evolutionary & environmental sciences

For a reference copy of the document with all sections, see nature.com/documents/nr-reporting-summary-flat.pdf

Life sciences study design

All studies must disclose on these points even when the disclosure is negative.

Sample size	No statistical methods were used to predetermine the sample sizes. The group sizes (n=3-12) were determined based on our experimental experience, which are indicated in each figure and were enough to facilitate the statistical analysis.
Data exclusions	No data were excluded.
Replication	Experiments were repeated at least three times and experimental findings were reproducible.
Randomization	All experimental samples or models were allocated randomly to each treatment group.
Blinding	In histological assessments, tissues were analyzed by an experienced investigator who was blinded as to treatment-group assignment. For other experiments, no blinded procedure was performed, since all the experiments were conducted at the same condition and data were collected using the same instruments that did not require scoring by subjective judgment.

Reporting for specific materials, systems and methods

We require information from authors about some types of materials, experimental systems and methods used in many studies. Here, indicate whether each material, system or method listed is relevant to your study. If you are not sure if a list item applies to your research, read the appropriate section before selecting a response.

Materials & experimental systems

n/a	Involved in the study	
☐	☒	Antibodies
☐	☒	Eukaryotic cell lines
☒	☐	Palaeontology and archaeology
☐	☒	Animals and other organisms
☒	☐	Human research participants
☒	☐	Clinical data
☒	☐	Dual use research of concern

Methods

n/a	Involved in the study	
☒	☐	ChIP-seq
☐	☒	Flow cytometry
☒	☐	MRI-based neuroimaging

Antibodies

Antibodies used

The following antibodies were used for immunofluorescent staining:
1. Mouse monoclonal to iNOS (Abcam, Catalogue #: ab49999, dilution: 1:200)
2. Rabbit polyclonal to Mannose Receptor (Abcam, Catalogue #: ab64693, dilution: 1:200)
3. Donkey anti-mouse IgG H&L (Alexa Fluor®488, Abcam, Catalogue #: ab150105, dilution: 1:200)
4. Donkey anti-rabbit IgG H&L (Alexa Fluor® 594, Abcam, Catalogue #: ab150076, dilution: 1:200)

The following primary antibodies were used for flow cytometry:
1. APC anti-mouse CCR7 (Biolegend, Clone: 4B12, Catalogue #: 120108, dilution: 1:100)
2. PE anti-mouse CD206 (Biolegend, Clone: C068C2, Catalogue #: 141706, dilution: 1:100)
3. APC anti-mouse CD45 (Biolegend, Clone: 30-F11, Catalogue #: 103111, dilution: 1:100)
4. FITC anti-mouse CD11b (Biolegend, Clone: M1/70, Catalogue #: 101205, dilution: 1:100)
5. PerCP/Cy5.5 anti-mouse F4/80 (Biolegend, Clone: BM8, Catalogue #: 123127, dilution: 1:100)
6. PE/Cy7 anti-mouse CD80 (Biolegend, Clone: 16–10A1, Catalogue #: 104733, dilution: 1:100)
7. APC anti-mouse CD80 (Biolegend, Clone: 16–10A1, Catalogue #: 104713, dilution: 1:100)
8. FITC anti-mouse CD11c (Biolegend, Clone: N418, Catalogue #: 117305, dilution: 1:100)
9. PE anti-mouse I-A/I-E (Biolegend, Clone: M5/114.15.2, Catalogue #: 107607, dilution: 1:100)
10. PE/Cy7 anti-mouse CD86 (Biolegend, Clone: GL-1, Catalogue #: 105013, dilution: 1:100)
11. FITC anti-mouse CD3 (Biolegend, Clone: 17A2, Catalogue #: 100203, dilution: 1:100)
12. APC/Cy7 anti-mouse CD8 (Biolegend, Clone: 53–6.7, Catalogue #: 100714, dilution: 1:100)
13. PerCP/Cy5.5 anti-mouse CD4 (Biolegend, Clone: GK1.5, Catalogue #: 100433, dilution: 1:100)

14. FITC anti-mouse CD19 (Biolegend, Clone: 6D5, Catalogue #: 115505, dilution: 1:100)
15. PE anti-mouse CD138 (Biolegend, Clone: 281-2, Catalogue #: 142503, dilution: 1:100)
16. PE anti-mouse Foxp3 (Biolegend, Clone: MF-14, Catalogue #: 126403, dilution: 1:100)
17. APC anti-mouse B220 (Biolegend, Clone: RA3-6B2, Catalogue #: 103211, dilution: 1:100)
18. PE anti-mouse IgD (Biolegend, Clone: 11-26c.2a, Catalogue #: 405705, dilution: 1:100)
19. FITC anti-mouse IgG (Biolegend, Clone: Poly4053, Catalogue #: 405305, dilution: 1:100)

The following antibodies were used for enzyme linked immunosorbent assay:
1. Mouse TNF-α ELISA Kit (Anogen, Catalogue #: MEC1003)
2. Mouse IL-1β ELISA Kit (Anogen, Catalogue #: MEC1010)
3. Mouse IL-6 ELISA Kit (Anogen, Catalogue #: MEC1008)
4. Mouse IL-12p70 Precoated ELISA Kit (Dakewei Biotech, Catalogue #: 1211202)
5. Mouse IgG ELISA Kit (Crystalchem, Catalogue #: 80644)
6. Mouse IgM ELISA Kit (Crystalchem, Catalogue #: 80651)

Validation

The antibodies used for immunofluorescent staining were verified by the supplier as below:
1. Mouse monoclonal to iNOS: https://www.abcam.cn/inos-antibody-nos-in-ab49999.html
2. Rabbit polyclonal to Mannose Receptor: https://www.abcam.cn/mannose-receptor-antibody-ab64693.html
3. Donkey anti-mouse IgG H&L: https://www.abcam.cn/donkey-mouse-igg-hl-alexa-fluor-488-ab150105.html
4. Donkey anti-rabbit IgG H&L: https://www.abcam.cn/donkey-rabbit-igg-hl-alexa-fluor-594-ab150076.html

The antibodies used for flow cytometry were verified by the supplier as below:
1. APC anti-mouse CCR7 (4B12): https://www.biolegend.com/en-us/products/apc-anti-mouse-cd197-ccr7-antibody-2822
2. PE anti-mouse CD206 (C068C2): https://www.biolegend.com/en-us/search-results/pe-anti-mouse-cd206-mmr-antibody-7424
3. APC anti-mouse CD45 (30-F11): https://www.biolegend.com/en-us/search-results/apc-anti-mouse-cd45-antibody-97
4. FITC anti-mouse CD11b (M1/70): https://www.biolegend.com/en-us/products/fitc-anti-mouse-human-cd11b-antibody-347
5. PerCP/Cy5.5 anti-mouse F4/80 (BM8): https://www.biolegend.com/en-us/products/percp-cyanine5-5-anti-mouse-f480-antibody-4303
6. PE/Cy7 anti-mouse CD80 (16–10A1): https://www.biolegend.com/en-us/products/pe-cyanine7-anti-mouse-cd80-antibody-9320
7. APC anti-mouse CD80 (16–10A1): https://www.biolegend.com/en-us/search-results/apc-anti-mouse-cd80-antibody-2340
8. FITC anti-mouse CD11c (N418): https://www.biolegend.com/en-us/products/fitc-anti-mouse-cd11c-antibody-1815
9. PE anti-mouse I-A/I-E (M5/114.15.2): https://www.biolegend.com/en-us/search-results/pe-anti-mouse-i-a-i-e-antibody-367
10. PE/Cy7 anti-mouse CD86 (GL-1): https://www.biolegend.com/en-us/search-results/pe-cyanine7-anti-mouse-cd86-antibody-3046
11. FITC anti-mouse CD3 (17A2): https://www.biolegend.com/en-us/search-results/fitc-anti-mouse-cd3-antibody-45
12. APC/Cy7 anti-mouse CD8a (53–6.7): https://www.biolegend.com/en-us/products/apc-cyanine7-anti-mouse-cd8a-antibody-2269
13. PerCP/Cy5.5 anti-mouse CD4 (GK1.5): https://www.biolegend.com/en-us/search-results/percp-cyanine5-5-anti-mouse-cd4-antibody-4220
14. FITC anti-mouse CD19 (6D5): https://www.biolegend.com/en-us/search-results/fitc-anti-mouse-cd19-antibody-1528
15. PE anti-mouse CD138 (281-2): https://www.biolegend.com/en-us/search-results/pe-anti-mouse-cd138-syndecan-1-antibody-7519
16. PE anti-mouse Foxp3 (MF-14): https://www.biolegend.com/en-us/search-results/pe-anti-mouse-foxp3-antibody-4660
17. APC anti-mouse B220 (RA3-6B2): https://www.biolegend.com/en-us/search-results/apc-anti-mouse-human-cd45r-b220-antibody-442
18. PE anti-mouse IgD (11-26c.2a): https://www.biolegend.com/en-us/search-results/pe-anti-mouse-igd-1379
19. FITC anti-mouse IgG (Poly4053): https://www.biolegend.com/en-us/products/fitc-goat-anti-mouse-igg-minimal-x-reactivity-1391

The following antibodies were used for enzyme linked immunosorbent assay:
1. Mouse TNF-α ELISA Kit: https://www.anogen.net/mouse-tnf-elisa-kit.html
2. Mouse IL-1β ELISA Kit: https://www.anogen.net/mouse-il-1-elisa-kit.html
3. Mouse IL-6 ELISA Kit: https://www.anogen.net/mouse-il-6-elisa-kit.html
4. Mouse IL-12p70 Precoated ELISA Kit: http://bioec.cn/r/1211202
5. Mouse IgG ELISA Kit: http://www.crystalchem.com/mouse-igg-elisa-kit.html
6. Mouse IgM ELISA Kit: https://www.crystalchem.com/mouse-igm-elisa-kit.html

Eukaryotic cell lines

Policy information about cell lines

Cell line source(s)	MC3T3-E1 pre-osteoblasts and RAW264.7 macrophages were obtained from Shanghai Institute of Cell Biology, Chinese Academy of Science.
Authentication	Cell lines were authenticated by STR profiling.
Mycoplasma contamination	No mycoplasma contamination was detected for all the cell lines.
Commonly misidentified lines (See ICLAC register)	No commonly misidentified cell lines were used.

Animals and other organisms

Policy information about studies involving animals; ARRIVE guidelines recommended for reporting animal research

Laboratory animals	Male BALB/c mice (6–8 weeks) were purchased from Laboratory Animal Management Department, Shanghai Family Planning Research Institute. Mice were housed in temperature-controlled (22±2°C) facilities with 12 h dark-light cycles and an average humidity rate between 40% and 70%.
Wild animals	The study did not involve wild animals.
Field-collected samples	The study did not involve samples collected from the field.
Ethics oversight	All of the animal experiments performed in this research were approved by the Institutional Animal Care and Use Committee of the Shanghai Sixth People's Hospital (DWLL2021-0755).

Note that full information on the approval of the study protocol must also be provided in the manuscript.

Flow Cytometry

Plots

Confirm that:

- [x] The axis labels state the marker and fluorochrome used (e.g. CD4-FITC).
- [x] The axis scales are clearly visible. Include numbers along axes only for bottom left plot of group (a 'group' is an analysis of identical markers).
- [x] All plots are contour plots with outliers or pseudocolor plots.
- [x] A numerical value for number of cells or percentage (with statistics) is provided.

Methodology

Sample preparation	The tissues were firstly mechanically dissected into small pieces, then digested with 1mg/mL collagenase IV and 100 µg/mL deoxyribonuclease at 37°C for 30 min. After filtered through a 70-µm filter, single cells were collected. Then, the single-cell suspension was treated with ACK lysis buffer for 5 min at room temperature to lyse RBCs. The single-cell suspension was incubated with anti-CD16/32 antibody for 10 min to block Fc receptors, and further incubated with fluorescent-labelled antibodies for 30 min on the ice. For intracellular staining, cells were fixed, permeablized and stained for intracellular antigens.
Instrument	BD LSRFortessa
Software	BD FACSDiva, FlowJo(v10)
Cell population abundance	No cell sorting was performed.
Gating strategy	Generally, cells were first gated on FSC/SSC. The cell populations within the gate were further analyzed based on expression of markers. Blank and single positive staining were used to determine the "true" positive and adjust compensation. Gating was then based on positive level. The detailed gating strategy could be found in Supplementary Information.

- [x] Tick this box to confirm that a figure exemplifying the gating strategy is provided in the Supplementary Information.
